# The Redox-Sensing Regulator Rex Modulates Central Carbon Metabolism, Stress Tolerance Response and Biofilm Formation by *Streptococcus mutans*

**DOI:** 10.1371/journal.pone.0044766

**Published:** 2012-09-13

**Authors:** Jacob P. Bitoun, Sumei Liao, Xin Yao, Gary G. Xie, Zezhang T. Wen

**Affiliations:** 1 Department of Oral and Craniofacial Biology, School of Dentistry, Louisiana State University Health Sciences Center, New Orleans, Louisiana, United States of America; 2 Biology and Bioinformatics, Los Alamos National Laboratory, New Mexico, United States of America; 3 Department of Microbiology, Immunology, and Parasitology, School of Medicine, Louisiana State University Health Sciences Center, New Orleans, Louisiana, United States of America; University of Kansas Medical Center, United States of America

## Abstract

The Rex repressor has been implicated in regulation of central carbon and energy metabolism in Gram-positive bacteria. We have previously shown that *Streptococcus mutans*, the primary causative agent of dental caries, alters its transcriptome upon Rex-deficiency and renders *S. mutans* to have increased susceptibility to oxidative stress, aberrations in glucan production, and poor biofilm formation. In this study, we showed that *rex* in *S. mutans* is co-transcribed as an operon with downstream *guaA*, encoding a putative glutamine amidotransferase. Electrophoretic mobility shift assays showed that recombinant Rex bound promoters of target genes avidly and specifically, including those down-regulated in response to Rex-deficiency, and that the ability of recombinant Rex to bind to selected promoters was modulated by NADH and NAD^+^. Results suggest that Rex in *S. mutans* can function as an activator in response to intracellular NADH/NAD^+^ level, although the exact binding site for activator Rex remains unclear. Consistent with a role in oxidative stress tolerance, hydrogen peroxide challenge assays showed that the Rex-deficient mutant, TW239, and the Rex/GuaA double mutant, JB314, were more susceptible to hydrogen peroxide killing than the wildtype, UA159. Relative to UA159, JB314 displayed major defects in biofilm formation, with a decrease of more than 50-fold in biomass after 48-hours. Collectively, these results further suggest that Rex in *S. mutans* regulates fermentation pathways, oxidative stress tolerance, and biofilm formation in response to intracellular NADH/NAD^+^ level. Current effort is being directed to further investigation of the role of GuaA in *S. mutans* cellular physiology.

## Introduction

*Streptococcus mutans*, the major causative agent of human dental caries, lives almost exclusively as tenacious biofilms on the tooth surface in an environment featured with fluctuating and often unpredictable conditions in nutrient availability, pH, temperature, oxygen tension, saliva, and shearing force [1], [2]. It has evolved multiple mechanisms to survive various kinds of environmental insults, optimize carbohydrate catabolism, and accumulate significantly on the tooth surface under certain conditions, as seen in carious sites [1], [3]. *S. mutans* is considered a homofermentative lactic acid bacterium, although the metabolic pathways of glucose by this bacterium vary, depending on the environmental conditions [4], [5], [6], [7]. When glucose is available in excess or during aerobic growth, *S. mutans* catabolizes glucose through homofermentation pathways, yielding primarily lactate. During growth under glucose limiting or anaerobic conditions, *S. mutans* undergoes heterofermentation, generating also formate, acetate, acetoin, and ethanol.

Despite the abundance of oxygen in the oral cavity, plaque biofilms support growth of a wide range of bacteria, including facultative and obligate anaerobes. Aerobes on the top of the plaque can quickly utilize the oxygen, lowering the redox potential. In fact, deep within the plaque, the environment is thought to be mainly anaerobic [8], [9]. Nevertheless, plaque bacteria, including anaerobes, have developed active defense mechanisms against oxygen and oxidative stress [10]. *S. mutans*, a facultative anaerobe, lacks catalase, cytochrome oxidases, and a complete electron transport chain [11], [12]. In *S. mutans*, oxygen and the deleterious reactive oxygen species (ROS) are scavenged through an array of cytoprotective enzymes, including alkylhydroperoxide reductases (AhpCF), glutathione reductase (GshR), superoxide dismutase (SodA), thioredoxin reductase (TrxA), and NADH oxidase (Nox) [11], [13], [14], [15].

*S. mutans* can utilize various carbohydrates at micro-concentrations [16], [17], [18], [19]. One crucial process for continued carbohydrates catabolism is the regeneration of NAD^+^ [10], [20]. Aerobically, with glucose in excess, the intracellular concentration of fructose 1,6-bisphosphate becomes elevated, and in turn, induces lactate dehydrogenase (Ldh) expression [21]. Increased Ldh expression maintains the redox poise needed for continued glycolytic activity since pyruvate is converted into lactate with the concomitant oxidation of NADH to NAD^+^. On the other hand, when glucose concentration is low, intermediary fructose 1,6-bisphosphate concentration is low and Ldh is not induced [21]. Under anaerobiosis, the bottleneck of catabolism is the re-oxidation of NADH to NAD^+^. Anaerobiosis induces the expression of pyruvate-formate lyase (Pfl), which can split pyruvate into formate and acetyl-CoA. The mechanism of Pfl does not directly impact the NADH:NAD^+^ ratio; however, the downstream action of alcohol-acetylaldehyde dehydrogenase (AdhE) can reduce acetyl-CoA into ethanol with the concomitant oxidation of NADH to NAD^+^. In *Staphylococcus aureus*, the shift to anaerobic conditions induced genes encoding exporters of the fermentation products lactate, formate, acetate, and 2,3-butanediol [22].

Rex is a member of the winged-helix family of DNA transcriptional repressors and plays a central role in regulation of energy metabolism by sensing the NADH:NAD^+^ ratio [23], [24]. The Rex family of proteins has been shown to optimize energy production through activation of genes poised to act more efficiently anaerobically [22], [23], [24], [25], [26]. In *S. aureus*, the shift from an aerobic to an anaerobic environment is associated with an increased glycolytic rate along with increased transcription of genes associated with lactate and formate export [22], [27]. High NADH:NAD^+^ ratios can be avoided by increasing transcription of pyruvate dehydrogenase and AdhE [25], [28].

Previously, we showed that Rex in *S. mutans* is involved in biofilm formation and oxidative stress tolerance [25]. This study was designed to elucidate members of the Rex regulon in *S. mutans* that contribute to the shift from homofermentation to heterofermentation and the oxidative stress tolerance. By combining protein-DNA interaction studies with RealTime-PCR and phenotype analysis, we have demonstrated that Rex can bind target promoters and regulate genes critical to the central carbon catabolism, oxidative stress tolerance, and biofilm formation. RT-PCR also revealed that *rex* in *S. mutans* is auto-regulated and co-transcribed with *guaA* (SMU.1054), for a putative glutamine amidotransferase. Deficiency of Rex and GuaA caused drastic defects in oxidative stress tolerance and biofilm formation.

## Materials and Methods

### Plasmids, bacterial strains, and growth conditions

Bacterial strains and plasmids used in this study are listed in Table 1. *S. mutans* strains were maintained in brain heart infusion (BHI) (Difco Laboratories) medium. Solid media were prepared similarly, but Bacto agar (Difco Laboratories) was added at a concentration of 1.5% (w/v). Kanamycin (1 mg mL^−1^) and/or spectinomycin (1 mg mL^−1^) were added to the growth medium, when needed. Unless stated otherwise, cultures were grown aerobically in a 37°C chamber containing 5% CO_2_ under static conditions. For growth studies, overnight cultures were diluted 1∶100 into fresh BHI and allowed to continue to grow. When reaching mid-exponential phase (optical density at 600 nm, OD≅0.4), cultures were diluted 1∶100 again and growth was then monitored using a Bioscreen C (Oy Growth Curves AB Ltd, Finland) at 37°C with and without sterile mineral oil overlay [25], [29]. The OD was monitored every 30 minutes after gentle shaking for 10 seconds. All *E. coli* strains were grown in Luria Bertani medium at 37°C aerobically with or without inclusion of kanamycin (40 µg/ml) and/or ampicillin (100 µg/ml).

**Table 1:** Bacterial strains and plasmids used in this study.

Strains/plasmids	Major properties	References
*S. mutans* UA159	wildtype, serotype c	[17]
*S. mutans* TW239	UA159 derivative, Δ*rex*, Kan^r^	[25]
*S. mutans* TW239C	TW239 carrying rex in pDL278, Kan^r^, Sp	[25]
*S. mutans* TW263	UA159 derivative, Δ*guaA*, Kan^R^	This study
*S. mutans* TW263C	TW263 carrying *guaA* in pDL278, Kan^r^, Sp^r^	This study
*S. mutans* JB314	UA159 derivative, Δ*rex*, Δ*guaA*, Sp^r^	This study
*S. mutans* JB315	JB314 complemented with *guaA*, Δ*rex*, Kan^r^, Sp^r^	This study
*S. mutans* JB316	JB314 complemented with *rex*, Δ*guaA*, Kan^r^, Sp^r^	This study
*E. coli* DH10B	Cloning host, Δ*mcrA*, Δ*mcrBC*, Δ*mrr*, and Δ*hsd*	Invitrogen
*E. coli* M15	Expression strain, carrying pRep4, Kan^r^	Qiagen, Inc.
pDL278	Shuttle vector, Sp^r^	[33]
pQE30	Expression vector, Amp^R^	Qiagen, Inc.

Note: Kan^r^, Sp^r^, and Amp^r^ are kanamycin, spectinomycin and ampicillin resistant, respectively.

### Construction of *S. mutans* mutants and complement strains

Strains deficient of GuaA were generated using a PCR-ligation-mutation strategy described elsewhere [30], [31] (Table 1). The resulting mutant, TW263, was further analyzed by PCR and DNA sequencing to verify the deficiency of target gene and sequence accuracy. Similarly, a Rex- and GuaA-double mutant, JB314, was generated by replacing a large portion of both *rex*- and *guaA*-coding sequences, from nucleotide 136 (from translational initiation site ATG) of *rex* to nucleotide 598 of *guaA*, with a non-polar spectinomycin resistant determinant [32]. TW239, a Rex-deficient mutant, and its complement strain, TW239C, were generated previously [25]. For *guaA* mutant complementation, the coding sequence of *guaA* plus the cognate promoter region was amplified by PCR with the genomic DNA of strain TW239 as the template using high fidelity Phusion DNA polymerase (New England BioLabs, Ipswich, MA). The resulting amplicon with *rex* deleted and replaced with a non-polar kanamycin resistance element of similar size was cloned into the streptococcal shuttle vector pDL278 [25], [29], [33], [34], and the resulting construct was used to transform TW263 to generate the complement, TW263C. For complementation of JB314 with *gua*A in single copy, the *guaA*-coding sequence and its cognate promoter region were amplified by PCR using primers 1053-55′ and 1054-33′ with genomic DNA of TW239 as the template (Table 2). The resulting mutants isolated from BHI with spectinomycin and kanamycin would restore *guaA* in JB314 at its natural locus, and were designated as JB315. Similarly, JB314 was transformed with the amplicon generated similarly using TW263 as the template, and derivative, JB316, isolated from BHI with spectinomycin and kanamycin plates would have *rex* phenotypes restored. *S. mutans* UA159, TW239, and TW263 were also transformed with the empty vector pDL278 to calibrate growth rates in the presence of spectinomycin.

**Table 2:** Primers used in this study.

Name	Nucleotide Sequence	Applications
Rex-Fw	Forward, 5′-GGTCGTGCTCTCCTGAATTATCG-3′	RT-PCR analysis
GuaA-Rv	Reverse, 5′-TTCGTGATTCAAGCAGCAGTTC-3′	RT-PCR analysis
148Fw	Forward, 5′-ATTTTTTTGTGAAAAGGGTTACAATGACAAG-3′	Rex-binding site #1 oligo
148Rv	Reverse, 5′-CTTGTCATTGTAACCCTTTTCACAAAAAAAT-3′	Rex-binding site #1 oligo
m148Fw	Forward, 5′-ATTTTTAACAGTAAAGGGTTACAATGACAAG-3′	Mutation of Rex-binding site #1 oligo
m148Rv	Reverse, 5′-CTTGTCATTGTAACCCTTTACTGTTAAAAAT-3′	Mutation of Rex-binding site #1 oligo
140Fw	Forward, 5′-TTATGATGTCATTATCATCG-3′	20 bp oligo within *gshR* promoter
140Rev	Reverse, 5′-CGATGATAATGACATCATAA-3′	20 bp oligo within *gshR* promoter
1363cFw	Forward, 5′-GATGATTTTTCGTCTATA-3′	18 bp oligo within *tpn* promoter
1363cRev	Reverse, 5′-TATAGACGAAAAATCATC-3′	18 bp oligo within *tpn* promoter
p1053Fw	Forward, 5′-GGCAAAGTAAAGTCTAACAGTGTTTA-3′	*rex* promoter amplification
p1053Rev	Reverse, 5′-TTATCGAAAGTCACTGTACTTCCTC-3′	*rex* promoter amplification
p148Fw	Forward, 5′-CCTTGGTACTTTATTGCTGATAAGAGT-3′	*adhE* promoter amplification
p148Rev	Reverse, 5′-TAGCTGCTTCTTCAGCAGTGAG-3′	*adhE* promoter amplification
p1410Fw	Forward, 5′-ACTAAGCAAACCTCCCATCTA-3′	*frdC* promoter amplification
p1410Rev	Reverse, 5′-ATGACAGTTCCTCGCATTT-3′	*frdC* promoter amplification
p1363Fw	Forward, 5′-GGTCTGGCATCAGATGG-3′	*tpn* promoter amplification
p1363Rev	Reverse, 5′-AGTCCTTAAGTAAAACCACATC-3′	*tpn* promoter amplification
p140Fw	Forward, 5′-ACAAGCCCATACTGAGTTTG-3′	*gshR* promoter amplification
p140Rev	Reverse, 5′-GGCTAGAGTCACACCTGAAACA-3′	*gshR* promoter amplification
p1115Fw	Forward, 5′-TCTGGAAGAGCCCGAGC-3′	*ldh* promoter amplification
p1115Rev	Reverse, 5′-CAGCATCCGCACAGTCT-3′	*ldh* promoter amplification
1054 55′	Forward, 5′-AGTTTAATACAGAGATTGGAGAG-3′	PCR of region 5′ to *guaA* for mutagenesis
1054 53′RI	Reverse, 5′-TGATATCGGGAATTCTAGCATGTG-3′	PCR of region 5′ to *guaA* for mutagenesis
1054 35′RI	Forward, 5′-TCTGAATTCACTTTTCTAGGTGTTC-3′	PCR of region 3′ to *guaA* for mutagenesis
1053 53′ Bm	Reverse, 5′-TCTAACTGTCGCTGGATCCATACC-3′	PCR for *rex::guaA* deletion/mutation
1054 35′ Hd	Forward, 5′-TCTAAGCTTACTTTTCTAGGTGTTC-3′	PCR for *rex::guaA* deletion/mutation
1053comp-5RI	Forward, 5′-ACAGAGAATTCTGGACTTAGAGTTGTC-3′	PCR of *rex* for complementation
1053comp-3Bm	Reverse, 5′-AGTCACTGTGGATCCTCCTTTTTC-3′	PCR of *rex* for complementation
1054comp-5Bm	Forward, 5′-AAATAAGGAGGATCCATGAATAAACC-3′	PCR of *guaA* for complementation
1054comp-3Hd	Reverse, 5′-ATAAGCTTCCTTCAGGTTCAAC-3′	PCR of *guaA* for complementation
5′-1053	Forward, 5′-ATGGATCCATCCCTAAGGCGACAATC-3′	For Rex expression
3′-1053	Reverse, 5′-TGACATAAGCTTATGGTGTATAAGAC-3′	For Rex expression
Biotin-*adhE*1 Fw	Forward, 5′-Biotin-TTTTGTGAAAAGGGTTACAATG-3′	“Hot” Oligo for Chemiluminescent EMSA
adhE1 Fw	Forward, 5′-TTTTGTGAAAAGGGTTACAATG-3′	“Cold” Oligo for Chemiluminescent EMSA
adhE1 Rev	Reverse, 5′-CATTGTAACCCTTTTCACAAAA -3′	“Cold” Oligo for Chemiluminescent EMSA
1053 55′	Forward, 5′-TCTAATGAAGATGCTCTTCCTC-3′	PCR for *rex::guaA* deletion & complementation
1054 33′	Reverse, 5′-TCTTATGTATTCAATCCATTCTGC-3′	PCR for *rex::guaA* deletion, complementation,

### Rex overexpression and purification

For Rex expression, the *rex* coding sequence was PCR amplified with the primers listed in Table 2, and then directly cloned in pQE30 (Qiagen, Inc.) following the manufacturer's instructions. *E. coli* DH10B (Invitrogen, CA) was used for plasmid amplification, and the insert was confirmed by DNA sequencing. The plasmid pQE-*rex* was transformed into expression host *E. coli* M15 (Qiagen, Inc.). The recombinant N-terminal His-tagged Rex protein (rRex) was expressed by IPTG (0.2 mM) induction and purified using a Ni-NTA column (Qiagen, Inc.) according to the manufacturer's instructions. Purified rRex was washed extensively with 20 mM Tris, pH 7.6, 250 mM NaCl, concentrated, and desalted. The concentration of Rex was determined using the extinction coefficient 10.32 mM^−1^ cm^−1^ while the concentration of NADH was determined using the extinction coefficient 6.3 mM^−1^ cm^−1^.

### Protein-DNA interactions

Protein-DNA interactions were analyzed using electrophoresis mobility shift assays (EMSA) similarly as described previously [32], [35]. The promoter regions of selected genes identified previously in Rex-deficient mutant by DNA-microarray analysis [25] were amplified by PCR from the primers listed in Table 2. Purified PCR products were incubated with increasing amounts of rRex in the reaction buffer containing 20 mM Tris pH 8.0, 1 mM EDTA, 75 mM KCl, 2 mM DTT, and 10% glycerol for 10 minutes at room temperature [22], [23], [24], [32]. When NAD^+^ or NADH was included for EMSA's, the samples were incubated for an additional 15 minutes upon the addition of NAD^+^ or NADH [22], [23], [24]. The reaction mixtures were separated on a 5% polyacrylamide gel under native conditions for 40 minutes and DNA mobility shift was visualized by SYBR Gold staining (Invitrogen, CA) using a Bio-Rad ChemiDoc XRS Imager [22]. For detailed dissection of the Rex-binding site (also Rex-box), oligonucleotides containing the putative Rex-binding site were synthesized, annealed, and then used as probes for EMSA (Table 2). Likewise, sets of oligos with mutations at particular nucleotides of the Rex-binding site were also used to determine Rex-binding specificity (Table 2). For cold competition assays, one oligonucleotide was 5′ biotinylated and annealed to its reverse complement. The cold probe was identical except it was not 5′ biotinylated. The EMSA reactions were carried out as described above. After electrophoresis, the gel was blotted to a Zeta™ Nylon membrane at 380 mA for 1 hour followed by UV cross-linking. Binding and the resulting mobility shift were visualized via LightShift Chemiluminescent EMSA Kit by following procedures recommended by the manufacturer (Pierce, Rockford, IL).

### Lactate Determination, End-point pH Analysis, and Glycolytic pH Drop

In an effort to identify the metabolic products of sugar catabolism, we measured and quantified the lactate generated by the wildtype and the mutant strains after 24 hour incubation in BHI by using EnzyChrom™ L-Lactate Assay Kit (Bioassay Systems, Inc.) [36]. In comparison, we monitored the end-point pH using a standard pH electrode (Orion, Inc.). To assay the glycolytic pH drop [37], we grew the strains to OD≅0.5, washed twice with ice cold de-ionized water, and resuspended the cells with 50 mM KCl and 1 mM MgCl_2_. The pH was adjusted to 7.2 with 0.1 M KOH before the addition of 50 mM glucose. The pH drop was monitored every minute for a period of 30 minutes.

### Biofilms formation

For biofilm formation, *S. mutans* strains were cultivated using a modified semi-defined biofilm medium with glucose (20 mM, BMG), sucrose (10 mM, BMS) or glucose (18 mM) and sucrose (2 mM) (BMGS) as the supplemental carbohydrate sources [25], [34], [38], [39]. For quantitative and structure analysis, biofilms were grown on hydroxylapatite discs in 12-well plates in BMGS and BMS for 24 hours and 48 hours, and were then stained with *Live/Dead Bac*Light fluorescent dye (Invitrogen, CA) before optical dissections using an Olympus Fluoview BX61 confocal laser scanning microscope at 600× magnification (Olympus) [25], [34]. At least 5 random areas were scanned for each sample. For post-acquisition analysis, simulated xyz three-dimensional images were generated using SLIDEBOOK 5.0 (Olympus) [25], [34]. Quantitative analysis, such as biovolume, biomass, surface area and mean height, was carried out using COMSTAT 2.0 with at least 5 independent scans for each sample [34], [40]. The biovolume is defined as the volume (µm^3^) of the biomass per µm^2^ of substratum area.

### Acid killing and hydrogen peroxide challenge assays

The abilities of the cells to withstand acid and hydrogen peroxide challenge were carried out as described previously [30], [34], [37]. Planktonic cultures were prepared from mid-exponential phase (OD_600 nm_≅0.5) cultures grown in BHI broth. For sessile populations, BMGS was used to support bacterial growth and glass slides were used as substratum [34], [41]. After 48 hours, biofilms were harvested using a sterile spatula, and following sonication to gently disperse the biofilms and detach the streptococcal chains, the cells were washed and then subjected to either acid killing in 0.1 M glycine, pH 2.8 or 0.1 M glycine pH 7.0 plus 0.2% hydrogen peroxide for the hydrogen peroxide killing assays [30], [34], [37]. Alternatively, methyl viologen (also paraquat, Sigma) at 10 mM or 25 mM was included in cultural medium, and the impact of superoxide generated on bacterial growth was monitored continuously using Bioscreen C.

### RNA extraction and RealTime-PCR analysis

For co-transcription and RealTime-PCR analysis, total RNA's were extracted from *S. mutans* strains prepared from either 50 mL of BHI broth or on glass slides grown in BMGS for 72 hours. Cells were collected by centrifugation at 4°C for 5 minutes, then immediately treated with RNAProtect (Qiagen, Inc.) as suggested by the manufacturer [34], [39], [41]. Following brief sonication to de-chain and disperse biofilms, cells were treated by glass beadbeater, and total RNAs were isolated using hot phenol as described previously [34], [39], [41]. To remove residual DNA, RNA preps were treated with DNaseI (Ambion, Inc.) and retrieved with the RNeasy purification kit (Qiagen, Inc.). For RealTime-PCR and co-transcription analysis, cDNA was synthesized with 0.25 µg of total RNA using the iScript cDNA synthesis kit (Bio-Rad) by following the procedures recommended by the manufacturer. RealTime-PCR was carried out with a Bio-Rad iCycler using procedures detailed elsewhere [34], [39], [41]. Primers used for co-transcription analysis are listed in Table 2 and those for RealTime-PCR in Table S1.

### Statistical Analysis

All quantitative data were further analyzed using paired student's *t*-test.

## Results

### The *rex* gene is co-transcribed with *guaA* and auto-regulated

**Figure 1:**
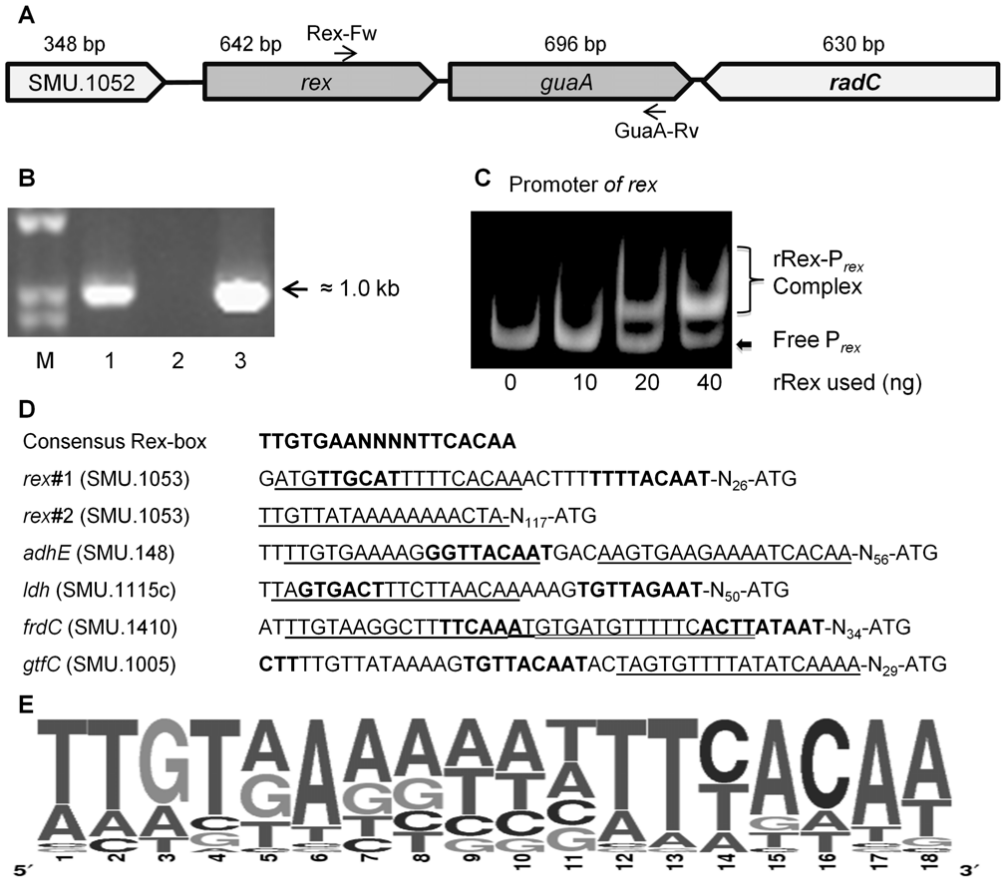
Schematic diagram of the *rex* flanking region and transcriptional analysis of the *rex* operon. (**A**) Schematic diagram of regions flanking *rex*, with the arrows indicating the direction of transcription and the numbers above indicating the sizes of the respective open reading frames in base pairs. Gene assignments and gene numbers above the diagram are based on Oralgen annotation. (**B**) RT-PCR analysis of *rex* operon. Following reverse transcription with iScriptase using primer GuaA-Rv, PCR amplification was performed with the primers Rex-Fw and GuaA-Rv, with RT products with and without iScriptase and genomic DNA as a positive control. Panel shows agarose gel electrophoresis of the PCR products. Lanes M, 1, 2, and 3 are MW marker, RT-PCR product, negative control without RT, and positive control with gDNA as a template. (**C**) EMSA analysis shows interaction of *rex* promoter with recombinant Rex. Inclusion of rRex resulted in mobility shift and such interaction was concentration-dependent. (**D**) Alignment of promoter regions of selected genes identified in TW239 that contain putative Rex binding sites. −10 and −35 regions as determined by BPROM program are in bold faces and putative Rex-binding sites are underlined or double-underlined in case where more than one site is identified. Positions of these elements were shown in numbers relative to start codon ATG of the respective genes. Consensus sequence was the conserved Rex-binding sites identified in *B. subtilis* and *S. aureus*. (**E**) Alignment of the putative and proven Rex-binding sites of the up-regulated genes in TW239. Promoters were scanned and aligned to the consensus Rex-binding site from *S. aureus* and *B. subtilis*. The indentified nucleotide sequences were then subjected to analysis using WebLogo (University of California, Berkeley) to generate the *S.* mutans consensus. Results showed that the Rex-binding site in *S. mutans* possesses more variability in the nucleotide composition than the other model organisms.

The *rex* gene (SMU.1053) of *S. mutans* is flanked by downstream *guaA* (SMU.1054), encoding a putative glutamine amidotransferase, and by the upstream SMU.1052, encoding a conserved hypothetical protein (www.oralgen.lanl.gov) (Fig. 1A). While there is a large intergenic region (295 bp) between SMU.1052 and *rex*, the gap between *rex* and *guaA* is only 23 nucleotides, suggesting these two genes may be co-transcribed. Computer based analysis using BPROM, a bacterial sigma70 promoter recognition program, identified a putative −10 and −35 element of the *rex* promoter situated 26 nucleotides upstream of the translational start site of Rex. Interestingly, both *rex* and *guaA*, but not SMU.1052, were found to be down-regulated in response to the deficiency of BrpA, a predicted surface-associated protein with global role in regulation of stress responses and biofilm formation in *S. mutans* [39], [42], [43]. To confirm that *rex* and *guaA* are transcriptionally linked, total RNAs were extracted from wildtype *S. mutans*, UA159, when the cultures were either grown planktonically in BHI (data not shown) and 3-day biofilms in BMGS. Reverse Transcription-PCR (RT-PCR) was carried out using reverse primer GuaA-Rv priming nucleotides 631–652 (relative to start codon ATG) of *guaA* (696 bp) and forward primer Rex-Fw priming nucleotides 286–308 within the 3′ end of *rex* (642 bp) (Fig. 1A, Table 2). As shown in Figure 1B, a single transcript of approximately 1.0 kb overlapping *rex* and *guaA* was amplified and identical in size to the PCR amplicon generated using genomic DNA as the template, further confirming that *guaA* is co-transcribed with *rex* under the conditions studied. In fact, our experimental evidence corroborates the bioinformatics data from Microbes Online (www. microbesonline.org/operons/gnc210007.html), which predicts *rex* and *guaA* to be the only genes within the *rex* operon. However, it cannot be ruled out that there are additional promoters driving the independent transcription of *rex* and *guaA* under certain conditions. The *radC* gene (SMU.1055) lies downstream of *guaA*, but is orientated in the opposite direction, signifying the end to the *rex* operon (Fig. 1A).

Computer-aided analysis of the *rex* promoter revealed two regions with high similarity to the consensus sequence of Rex-binding site that have been identified in *Streptomyces coelicolor, Bacillus subtilis* and *S. aureus*, TTGTGAAN_4_TTCACAA [44] (Fig. 1D). In fact, one of the putative Rex-binding sites, rex#1, actually overlaps the −35 element, providing an explanation as to how Rex feedback auto-regulation could occur (Fig. 1D). EMSA was then carried out using either the PCR-amplified promoter region of *rex* or oligonucleotides specific to the putative binding site (Table 2). As shown in Figure 1C, rRex was able to bind to the promoter sequence of *rex*, which further suggests that Rex is poised for auto-regulation. Consistently, our previous DNA microarray and RealTime-PCR data support the finding that the *rex-guaA* operon is auto-regulated. The *guaA* gene was up-regulated by approximately 5-fold in TW239, which had the *rex*-coding sequence deleted and replaced by a non-polar kanamycin resistance element [25], [29], [34].

### Binding of Rex to the target promoters is modulated by NADH:NAD^+^

**Figure 2:**
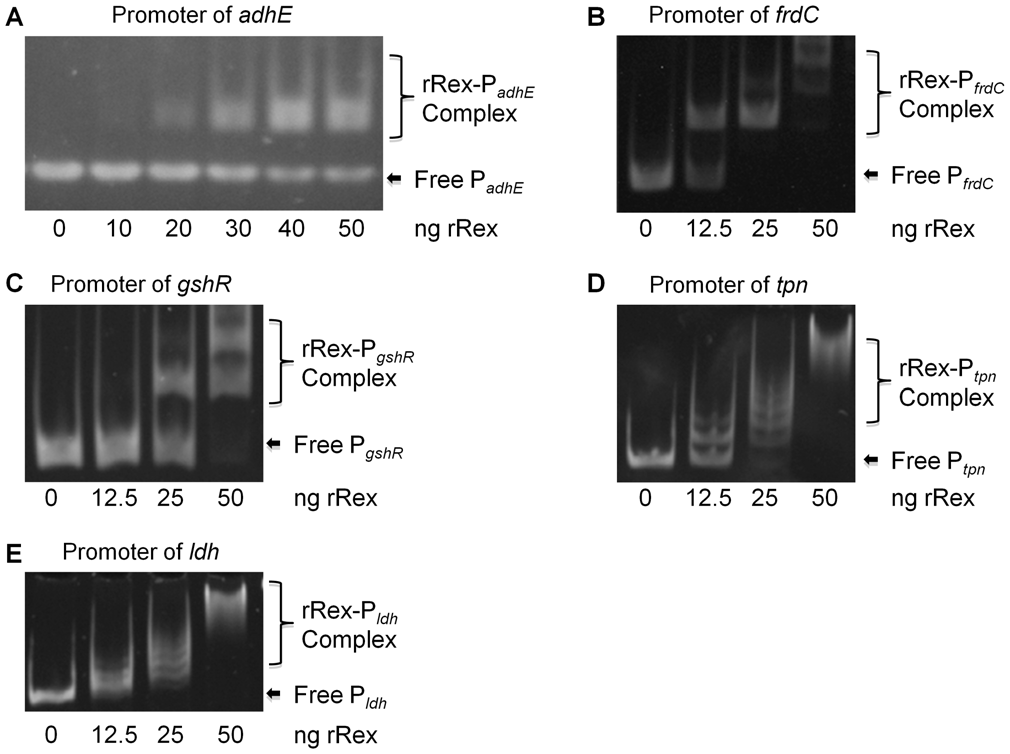
EMSA analysis with selected promoters. Promoters of selected genes were mixed with rRex and the impact of the presence of Rex on mobility was visualized via SYBR Gold staining. Data presented here show that rRex specifically binds the promoters of *adhE* (**A**), *frdC* (**B**), *gshR* (**C**), *tpn* (**D**), and *ldh* (**E**), respectively, and when added at different concentrations, causes mobility shift as a result of formation of rRex-promoter complexes.

Recent studies have shown that repressor Rex functions as a dimer and that binding to the operators of its target genes is modulated in response to the cellular NADH/NAD^+^ level [22], [23], [24], [44]. To verify the direct role of Rex in the regulation of the altered genes, we used EMSA to assess the interactions between rRex and the promoters of selected genes identified from DNA microarray analysis of TW239 [25]. We tested the promoters of two up-regulated genes in *adhE* (SMU.148) and *frdC* (SMU.1410 for fumarate reductase) and two down-regulated genes in *gshR* (SMU.140 for glutathione reductase) and *tpn* (SMU.1363 for a putative transposase). As seen in Figure 2, all of the promoters tested displayed a dose-dependent mobility shift to higher molecular mass of rRex-promoter complexes. For the *adhE* promoter, oligo of the Rex-binding sites were also chemically synthesized, and as shown by EMSA, they were able to bind to rRex (Fig. 2A). As expected in a cold competition assay, mobility shift was decreased with increasing amount of unlabeled DNA probe included in the EMSA reactions (Fig. S1, B). In addition, mutations of nucleotides that are considered critical within the Rex-box abolished rRex binding (Fig. S1, A). Consistently, no apparent mobility shift was observed when rRex was mixed with the promoter of *levD*, which is not regulated by Rex and whose promoter does not have an apparent Rex-box (Fig. 1S, C). These results further suggest the binding of rRex to the Rex-box of *adhE* promoter is nucleotides-specific.

**Figure 3:**
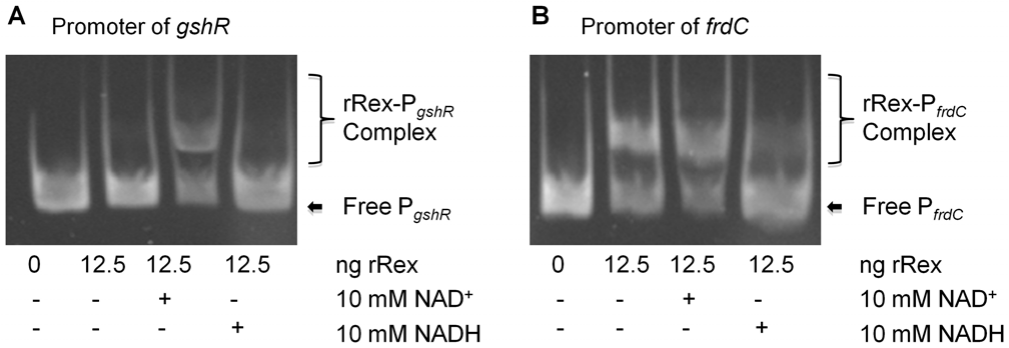
rRex binding with and without the inclusion of NAD^+^ and NADH. (**A**) rRex interactions with the promoter of *gshR* in the presence of 10 mM NAD^+^ or 10 mM NADH. The binding affinity between rRex and the *gshR* promoter is enhanced in the presence of NAD^+^ while the presence of NADH inhibits complex formation. (**B**) rRex interactions with the promoter of *frdC* in the presence of 10 mM NAD^+^ or 10 mM NADH. At low concentrations, rRex binds better to the repressor site of *frdC* when compared to *gshR* (A). Inclusion of NAD^+^ did not change the mobility pattern; however, NADH causes the complex to dissociate.

Recent studies in *B. subtilis* and *S. aureus* have shown that Rex's ability to bind to the promoters is dependent upon the NADH:NAD^+^ ratio. When NADH>NAD^+^, Rex dissociates from the promoter, resulting in alleviation of Rex-mediated repression and transcription of Rex-regulated genes [22], [23], [24], [26], [45]. To evaluate how nicotinamide moieties may affect Rex activity in *S. mutans*, we also examined the interactions of rRex with target promoters with inclusion of NAD^+^ and NADH in EMSA assays. In the EMSA pre-reaction, the target promoter of *gshR* and rRex were pre-incubated for 10 minutes, and then NADH and/or NAD^+^ (10 mM, final conc.) were added. As shown in Figure 3A, in the presence of NAD^+^, rRex bound to the promoter of *gshR*, as evidenced by the mobility shift. On the other hand, inclusion of NADH inhibited the formation of Rex-promoter complex. Alternatively, rRex bound the promoter of *frdC* under the same conditions even without any addition of exogenous NAD^+^ (Fig. 3B). The presence of NAD^+^ did not have major effect on mobility shift, but addition of NADH almost abolished such a binding activity (Fig. 3B). Effort was made to further evaluate the effect of NADH/NAD^+^ when mixed at different ratios on Rex-DNA binding efficiency, but the results were not evident as expected (data not shown), suggesting other factor(s) may be involved in Rex-mediated regulations in *S. mutans*.

**Figure 4:**
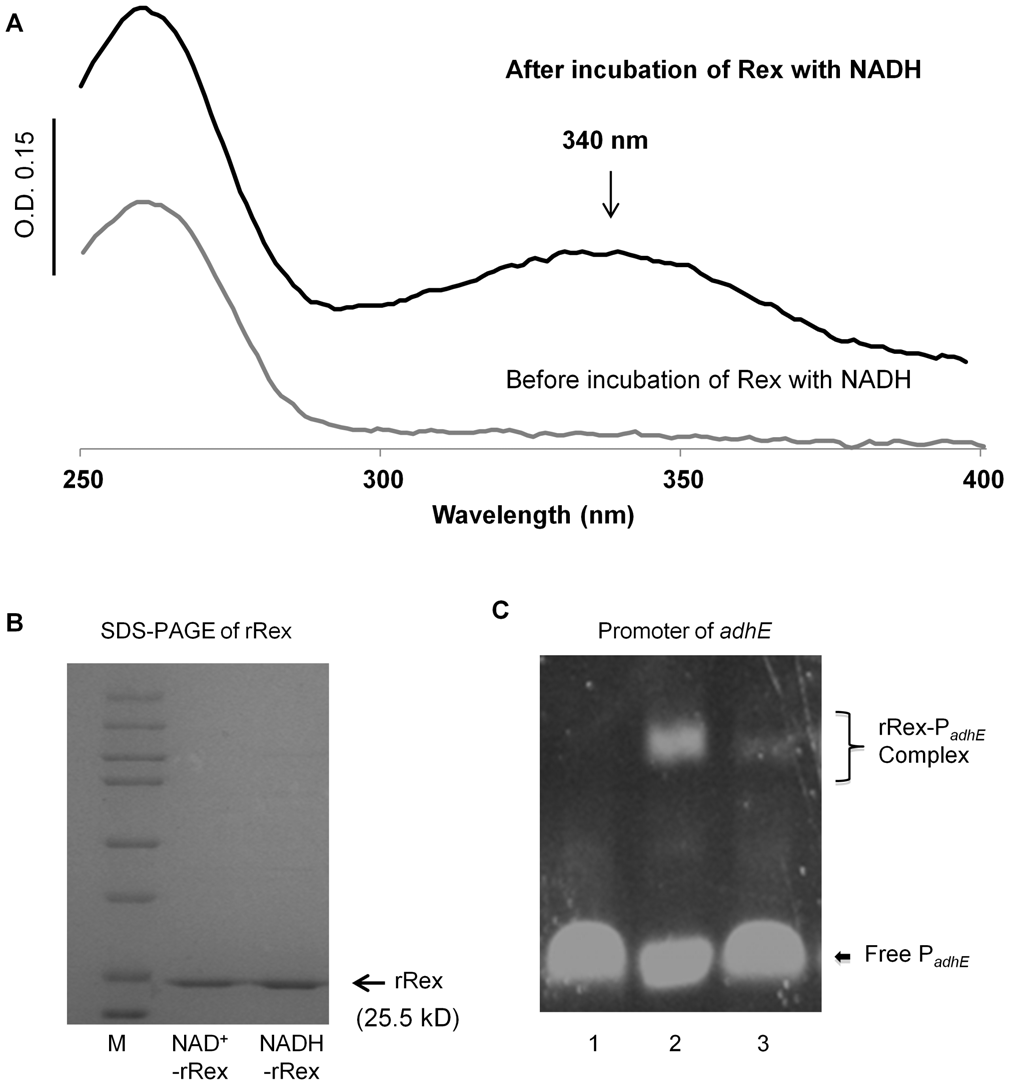
*In vitro* reconstitution of Rex with NAD^+^ and NADH. (**A**) The UV-Vis spectra of rRex after purification (light line). Incubation of rRex with 20-fold excess NADH (dark line) overnight at 4°C generates a different UV-Vis spectrum with a spectral peak at 340 nm, indicative of NADH binding to rRex. (**B**) SDS-PAGE after rRex reconstitution with either NAD^+^ or NADH verifies the presence of rRex. (**C**) EMSA analysis with the *adhE* promoter with NAD^+^-loaded (lane 2) and NADH-loaded (lane 3) rRex, or rRex as a negative control (lane 1). No additional NAD^+^ or NADH was included in the EMSA reactions.

Studies in *B. subtilis* suggest that Rex binds NADH and NAD^+^ tightly, with estimations of a *K_D_* lower than 100 nM for NADH [45]. Based on this evidence, it could be envisioned that Rex is always bound to either NADH or NAD^+^. However, after purification of rRex, there was minimal NADH or NAD^+^ bound, which is likely an artifact of the over-expression and purification processes. Thus, in an effort to fully reconstitute rRex with either NAD^+^ or NADH to mimic the intracellular conditions, rRex was incubated with 20-fold excess of NAD^+^ or NADH overnight on ice, and then desalted using a Centricon (Millipore) to remove free NAD^+^ or NADH (Fig. 4A). As seen in Figure 4A, the UV-Vis spectrum of purified rRex (grey line) does not show the NADH associated shoulder peak at 340 nm. Indeed, full reconstitution of rRex with NAD^+^ did not change the UV-Vis spectrum. However, after reconstitution of rRex with NADH, the UV-Vis spectrum shows the typical 340 nm peak attributed to the absorbance of NADH (black line), confirming that NADH is directly bound to rRex. SDS-PAGE analysis was carried out to confirm that rRex itself was maintained and intact in the samples of the reconstitutions (Fig. 4B). The reconstituted samples were used to carry out the EMSA reactions with the promoter of *adhE* without the addition of exogenous nicotinamide. As seen in Figure 4C, NAD^+^-loaded rRex binds to the promoter of *adhE*, and NADH-loaded rRex is inhibited in binding to the *adhE* promoter.

### Rex regulates heterofermentation and its deficiency affects the culture pH

**Figure 5:**
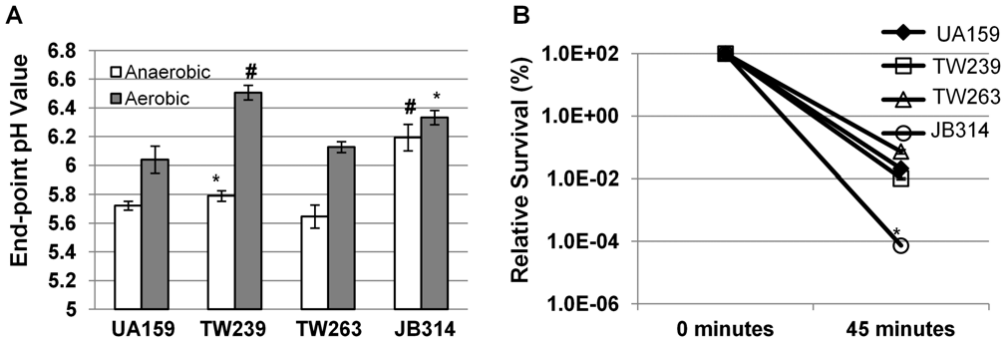
End-point pH analysis and acid killing. (**A**) Panel shows results of End-point pH analysis of *S. mutans* wildtype, UA159 and mutants deficient of Rex (TW239), GuaA (TW263) and both Rex and GuaA (JB314) grown in BM with glucose (18 mM) and sucrose (2 mM) under aerobic (grey bars) and anaerobic conditions (open bars), with * and # indicating significant differences between the wildtype and the particular mutants under the same conditions at a level of *P*<0.05 and *P*<0.001, respectively. Rex-deficiency increases the final resting pH, especially under aerobic conditions. (**B**) Panel illustrates survival rates of UA159 (diamond), TW239 (square), TW263 (triangle) and JB314 (circle) after acid -killing at pH 2.8 for 45 minutes. Symbols *, indicating significant difference between UA159 and JB314 at the level of *P*<0.01. The Rex/GuaA double mutant is approximately 2-logs more susceptible to acid than UA159.

It is apparent that Rex in *S. mutans* plays a major role in modulation of carbohydrates and energy metabolism. As shown previously by DNA microarray analysis and further confirmed by RealTime-PCR in this study [25] (Table 3), at least 17 genes with putative functions in carbohydrates metabolism were differentially regulated in response to Rex-deficiency. Of the genes up-regulated in TW239, several are known to be involved in heterofermentative catabolism [4], [6], [26], including *pfl* (SMU.402), *adhE*, *pdh* (SMU.1421/4 for pyruvate dehydrogenase complex), *frdC* and *adhABCD* (SMU.127-30 for the acetoin dehydrogenase complex) [25]. The commonality among these enzymes is that they all function to maintain the NADH:NAD^+^ ratio needed to reposition the cells for re-entry into glycolysis. In response to carbohydrate limiting and/or high intracellular NADH/NAD^+^ level, the bacterial cells may divert the catabolic pathways away from homofermentation towards heterofermentation, resulting in a decreased production of acids, especially lactic acid by *S. mutans*. To this effect, we sought to determine the effects of Rex-deficiency on end-point pH and lactate production after 24 hours of growth and evaluate the glycolytic rate via pH drop experiments. As seen in Figure 5A, we noticed significant increases in the end-point pH of the Rex-deficient mutant, TW239, especially during growth under aerobic conditions. When grown in BMGS, the average end-point pH for UA159 was 6.04 (±0.03), as compared to 6.51 (±0.05) (*P*<0.001) for TW239, 6.13 (±0.04) (*P*>0.05) for GuaA-deficient mutant, TW263, and 6.33 (±0.05) (*P*<0.05) for the Rex-deficient and GuaA-deficient double mutant, JB314, respectively. EnzyChrom™ L-Lactate Assay also showed a reduction of approximately 1.5 mM in lactate production as a result of Rex-deficiency, with average of 24.21 (±0.55) mM) for TW239 vs 25.74 (±0.66) mM for UA159 (Fig. S2). In addition, pH drop experiments showed that TW239 had a slow pH drop upon initiation by addition of glucose and maintained a baseline threshold of pH 4.10, as compared to the baseline threshold of pH 3.80 for UA159 after 20 minutes (Fig. S3). As expected, complementation of the mutant with *rex* in pDL278 shuttle vector was able to partly restore these phenotypes to the wildtype levels (Fig. S2, S3). The differences between TW239 and UA159 in end-point pH, lactate production, and glycolytic rates could be attributed at least in part to the increases in ethanol and acetoin production as a result of the increased expression of AdhE and the acetoin dehydrogenase complex AdhABCD in response to Rex deficiency [25], [46]. Similar trends were also observed when the strains were grown in BMS, although the differences were not as pronounced as in BMGS (data not shown). However, the exact metabolite pools await further investigation.

**Table 3:** RealTime-PCR analysis of selected genes.

Gene name	Locus	Description and putative function#	UA159*	TW239*	Fold-change@	*P*-value
16S rRNA	rRNA-16S	16S ribosome RNA	5.88E+07	5.68E+07	−1.04	0.62
*frdC*	SMU.1410	Fumarate reductase	7.16E+04	2.45E+05	3.46	0.001
*adhA*	SMU.127	Acetoin dehydrogenase	3.32E+06	9.04E+06	2.72	0.05
*mleS*	SMU.137	Malolactic enzyme	8.32E+05	1.48E+05	−5.61	0.05
*mleP*	SMU.138	Malate permease	2.69E+05	3.27E+04	−8.24	0.05
*dprA*	SMU.1001	DNA processing protein	1.50E+03	2.49E+03	1.67	0.02
*gor (gshR2)*	SMU.838	Glutathione reductase	2.00E+05	8.04E+04	−2.49	0.05
*gshR*	SMU.140	Glutathione reductase	8.72E+08	4.44E+07	−19.64	0.01
*nox*	SMU.1117c	NADH oxidase (H_2_O forming)	6.08E+05	2.80E+05	−2.67	0.01
*ahpC*	SMU.764	Alkyl hydroperoxide reductase (C)	7.75E+05	4.17E+05	−1.86	0.01

Note:

# Description and putative function of the selected genes are based upon the published *S. mutans* database.

* , The levels of expression are presented as copy numbers of the respective transcripts per µg of total RNA.

@ , Fold-change is defined as level of expression in the Rex-deficient mutant TW239 relative to those of the wildtype UA159, with a – representing down-regulation.

### Rex- and GuaA-deficiency affects acid- and oxidative stress responses

Previously, Rex-deficiency in *S. mutans* was shown to cause defects in oxidative stress tolerance, but not in acid tolerance response [25]. When assessed by acid-killing at pH 2.8, TW239 was found slightly more susceptible to acid killing than UA159, although the differences were not statistically significant (P>0.05), consistent with our previous findings [25]. Unlike TW239, the survival rate of the GuaA-deficient mutants, TW263, was slightly increased, but again the differences were not statistically significant from UA159 (Fig. 5B). However, deficiency of both Rex and GuaA in JB314 was shown to cause drastic increases in vulnerability to acid stress, with a reduction of survival rate in JB314 by more than 2-logs when compared to wildtype, UA159 (*P*<0.01). Complementation of this double mutant with *rex* plus its cognate promoter in JB316 and with *guaA* and its cognate promoter in JB315 were able to partly restore the phenotype to a level similar to TW263 and TW239, respectively (data not shown).

**Figure 6:**
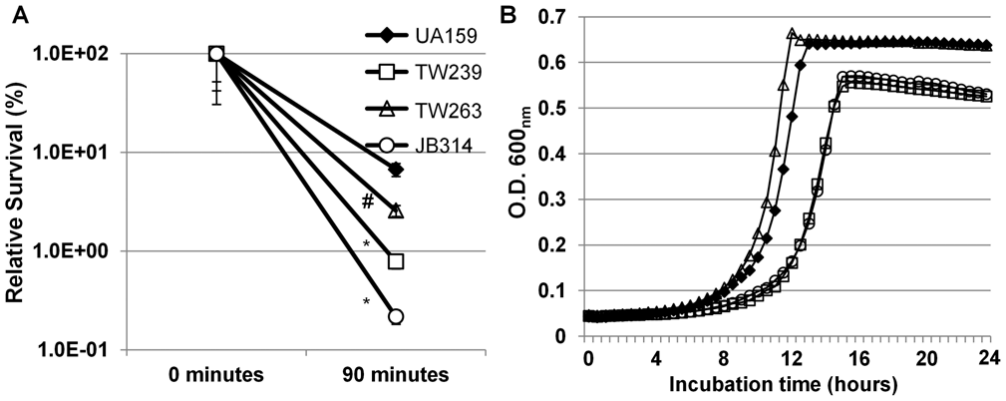
Hydrogen peroxide killing and methyl viologen challenge assays. (**A**) Panel shows survival rates of *S. mutans* UA159 (diamonds), TW239 (squares), TW263 (triangles) and JB314 (circles) biofilms after hydrogen peroxide treatment for 90 minutes at room temperature. Biofilms were grown in 50 mL tubes on sterile glass slides in BMGS. Symbols # and * indicate differences between UA159 and the particular mutant at significant level of *P*<0.01 and 0.001, respectively. (**B**) Panel B shows growth curves of *S. mutans* strain grown in BHI in the presence of 10 mM methyl viologen (also paraquat). Strain labels are the same as in panel (A).

Hydrogen peroxide killing assays were used to evaluate the impact of GuaA-deficiency on oxidative stress tolerance. Consistent with previous findings [25], TW239 was shown to be more susceptible to hydrogen peroxide killing than UA159, with a reduction in survival rate of more than 1-log (P<0.001), when compared to UA159 (Fig. 6A). When subjected to hydrogen peroxide killing, a reduction of more than 6-fold in survival rate was also measured with TW263 (P<0.001) when compared to UA159 (Fig. 6A). The survival rate of JB314, was further reduced by almost 2-logs, relative to UA159, which suggests an additive effect of Rex- and GuaA-deficiency on the susceptibility of JB314 to hydrogen peroxide. Consistently, when the strains were grown in the presence of methyl viologen (10 mM, final concentration), both TW239 and JB314 displayed greater challenge to methyl viologen than UA159 (Fig. 6B). Again, possession of a wildtype copy of the deleted gene plus the cognate promoter region in shuttle vector was able to partly complement the Rex-deficiency in TW239 (data not shown).

### Rex- and GuaA-deficiency causes major defects in biofilm formation

**Figure 7:**
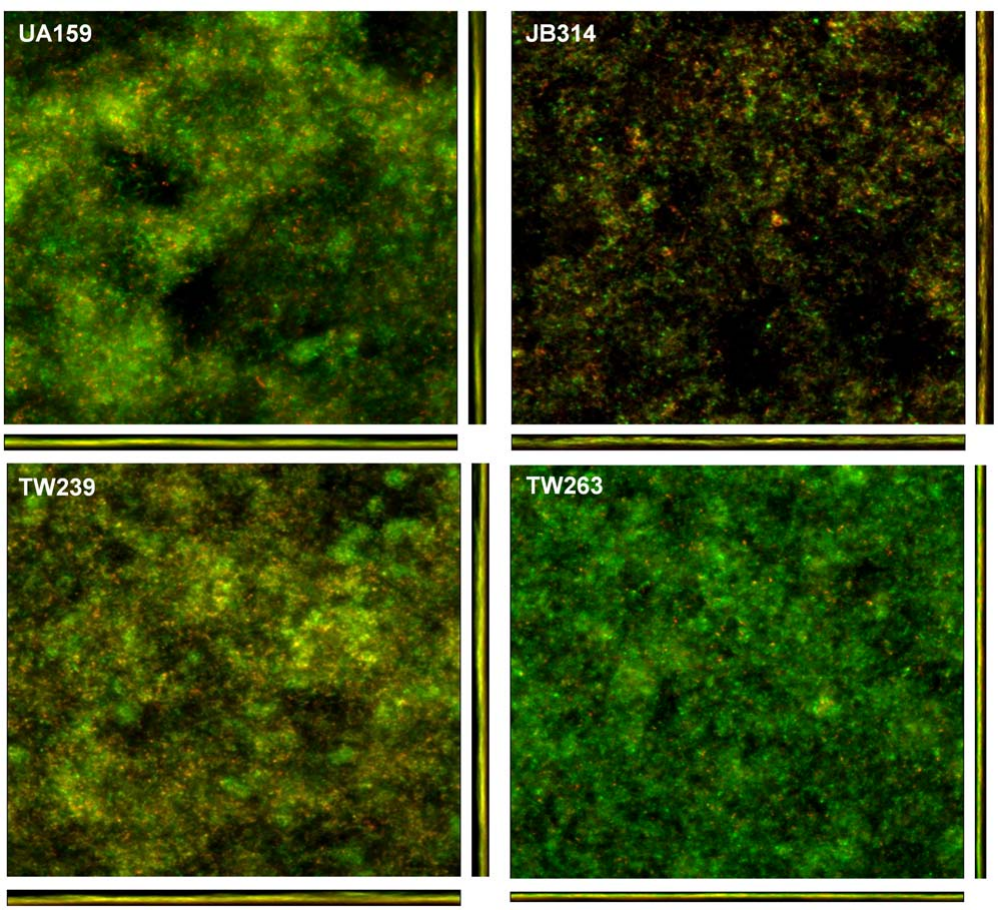
Biofilm analysis by confocal laser scanning microscope. *S. mutans* biofilms were grown anaerobically on hydroxylapatite discs in 12-well plates in BM medium supplemented with 10 mM sucrose for 48 hours. Following proper staining using LIVE/DEAD *Bac*Light fluorescent dye, biofilms were subjected to optical dissection using an Olympus laser scanning confocal microscope. Images were taken at 600× using a water immersion objective. Post-acquisition analyses were performed using SLIDEBOOK 5.0 (Olympus) and COMSTAT 2.0. Data presented here are representative xyz, xz and yz images (512×512) of UA159, TW239, TW263 and JB314 from more than three independent experiments.

Previous studies showed that deficiency of Rex in *S. mutans* had no major effect on growth of the Rex-deficient mutant, but altered biofilm formation, especially when grown in sucrose-containing medium [25]. Similarly, neither deficiency of GuaA alone nor both Rex and GuaA had any major effects on growth rates of the deficient mutants during growth in BHI and BMG (data not shown). To more fully evaluate the role of the *rex* operon in biofilm formation, biofilms of the Rex- and GuaA-deficient single mutants, TW239 and TW263, and the double mutant, JB314, were grown in BMS and BMGS on hydroxylapatite discs in 12-well plates simulating a static growth model under both aerobic and anaerobic conditions. Following Live/Dead *Bac*Light fluorescent staining, biofilms were analyzed using a confocal microscope equipped with an Olympus LUMFLN 60× water immersion objective [25], [34], [39]. When grown under anaerobic conditions on hydroxylapatite discs in BMS, wild-type UA159 formed robust biofilms after 48 hours (Fig. 7). Similar to UA159, TW263 also developed mostly viable biofilms after 48 hours, but unlike UA159, the biofilms of TW263 were flat and thin. When analyzed by COMSTAT, the bio-volume of TW263 biofilms was nearly half of that of UA159, with an average of 4.98 (±3.35) µm^3^/µm^2^ for TW263 vs. 9.10 (±3.54) µm^3^/µm^2^ for UA159, suggesting impaired growth of GuaA-deficient mutant in biofilms. Similar to our previous data, TW239 formed loose biofilms with porous structure, averaging a biovolume of 10.68 (±2.32) µm^3^/µm^2^ (P<0.001). However, relative to UA159 and the respective single mutants, biofilm formation by the Rex/GuaA-double mutant, JB314, were severely retarded during growth in BMS, accumulating an average biomass of just 0.21 (±0.18) µm^3^/µm^2^ (P<0.001) after 48 hours. Unlike the biofilms of UA159 and the respective single mutants, the biofilms of JB314 were mostly dead, showing red fluorescence after Live/Dead staining. Similar but less dramatic differences were observed between the different strains when grown under in the presence of glucose (Fig. S4).

## Discussion

A highly conserved transcriptional regulator, Rex has been implicated in regulation of key enzymes in carbon and energy metabolism [22], [23], [24], [45]. Previously, we showed that deficiency of Rex in *S. mutans* causes substantial alterations in the transcriptional profile and the abilities of the deficient mutant to survive oxidative stress and form biofilms [25]. Here, we provided further evidences that similar to *B. subtilis* and *S. aureus*, Rex in *S. mutans* also binds to target promoters and its binding efficiency is affected by the level of NADH:NAD^+^. Like *S. coelicolor*, but unlike *B. subtilis* [23], [24], Rex in *S. mutans* is subjected to Rex-mediated auto-regulation, a feedback mechanism commonly used to optimize the efficiency of cellular functions. These results further suggest that Rex in *S. mutans* plays a major role in modulation of the efficiency of carbon central metabolism and balance of the redox status.

Intensive studies mostly in *B. subtilis* and *S. aureus* have shown that repressor Rex binds to the target promoters as a dimer [22], [23], [24], [45]. In *B. subtilis*, Rex binds to a region of 5′-WWTGTGAANTNNTNNNCAAW-3′, where “W” denotes either A or T [23], [45]. In *S. aureus*, the consensus sequence is 5′-TTGTGAAWWWWTTCACAA-3′ [22]. During the preparation of this manuscript, Ravcheev *et al.* also used comparative genomics approach to infer candidate Rex DNA-binding motifs, and found that the DNA-binding motifs of Rex orthologs from 11 taxonomic groups showed significant conservation with a generalized consensus of TTGTGAANNNNTTCACAA [44]. Similarly, computer-aided analysis of the promoter regions of the de-repressed genes in *S. mutans* revealed region(s) with high similarity to the consensus sequence of the Rex-binding site deduced from other model microorganisms (Fig. 1D, 1E). EMSA analysis of selected promoters and/or synthetic oligos of the Rex-binding sites also demonstrated nucleotide-specific interactions with the recombinant Rex (Fig. 2–3, S1). However, further analysis of these promoter regions/Rex-binding sites also showed that differences exist between different promoters in nucleotide composition and number of Rex-binding sites (Fig. 1D, 1E). Consistently as seen in Figures 2 and 3, the promoters tested have also displayed differences in binding affinities for rRex, which signifies a hierarchal importance of Rex regulated genes. Indeed, our microarray data showed that Rex-deficiency effects transcription dissimilarly, suggesting either the involvement of other regulator(s) and/or tighter regulation depending on the nucleotide composition of the Rex-binding site. Two Rex-binding sites could be identified in the promoter of *rex* and *adhE* (Fig. 1D), although the exact significance of such possessions in Rex-mediated regulation remains unclear. Alignment of 41 proven and putative Rex-binding sites showed that the Rex-binding sites in *S. mutans* are similar to the established consensus and is more similar with those of *B. subtilis* (Fig. 1E) [23], [44], [45].

It is also worth noting that when using the consensus sequence to do a whole genome search allowing two or three mismatches, many more putative Rex-binding sites could be identified in intergenic regions and upstream of genes that were not identified in the TW239 microarray (data not shown), including lactate dehydrogenase gene *ldh* [25]. Unlike *S. aureus* [22], but similar to *B. subtilis* [22], [26], *ldh* was not identified by DNA microarray analysis in TW239. Further analysis by RealTime-PCR also revealed no significant difference between UA159 and TW239 during growth under similar conditions (data not shown). As shown in Figure 1D, one putative Rex-binding site was positioned at nucleotide 64 relative start codon ATG, and when analyzed by EMSA, rRex was indeed able to bind to this region (Fig. 2E). BHI is a rich medium but contains only 11.05 mM dextrose. Thus, it is possible that the conditions used to grow these cultures were not optimal for Ldh expression and that other regulatory mechanisms are likely involved in the Ldh expression. Nevertheless, these results further suggest that a broader role exists for Rex in regulation of *S. mutans* physiology under different conditions, although details on the functions and regulation of these genes await further investigation.

Previously identified by DNA microarray analysis [25] and further confirmed by RealTime-PCR in this study (Table 3), Rex-deficiency in *S. mutans* also causes down-regulation of more than 32 genes [25], a preliminary indication that Rex may also act as a transcriptional activator. In a recent study of a Ldh-deficient *E. faecalis* [47], Mehmeti *et al* also reported that Rex-binding sites were not only identified in many up-regulated genes, but also some of the genes that were down-regulated in the Ldh-deficient mutant. It was then suggested that Rex in *E. faecalis* may function as an activator, although no other information was provided concerning the relationship between these altered genes and Rex. In an effort to pinpoint the putative binding site for activator Rex, a 95 bp promoter region of *gshR* and a 410 bp promoter region of *tpn*, were amplified by PCR and then subjected to EMSA analysis. As shown in Figures 2 and 3, both promoter regions bound well to rRex. Similarly, inclusion of NAD^+^ enhanced the protein-DNA interactions, while NADH decreased the bindings. These results further suggest that Rex in *S. mutans* actually binds to the promoter of *gshR* and *tpn*, modulating their expression as an activator in response to the NADH/NAD^+^ level and probably other factors, although the binding site for activator Rex appears to be different from that of the repressor Rex. Studies are underway to dissect the binding sites for Rex as an transcriptional activator.

*S. mutans* possesses the molecular weaponry to target bursts of oxidative and nitrosative stress [10]. Reactive oxygen/nitrogen species (ROS/RNS) can be mitigated through small molecules, including free thiols, glutathione, ascorbate, and tocopherol or through enzymatic proteins and transcriptional regulators. Of the genes identified in TW239 by DNA-microarray analysis, several were known to play a role in oxidative stress tolerance, including the *mleSP* and *gshR* [25], [48]. The malolactic fermentation system, including malolactic enzyme MleS and malate permease MleP, functions in malate decarboxylation, yielding lactate, CO_2_ and ATP [49]. It is also recently shown to play a major role in *S. mutans* to protect from starvation and acid- and oxidative stress [48], [49]. Glutathione reductase (GshR) is well documented for its role in ROS detoxification in both eukaryotes and Gram-negative bacteria, and similar results were recently reported in *S. mutans* [50]. *S. mutans* possesses two homologues, *gshR* and *gshR-2* (or *gor*) by SMU.838 (www.lan.oralgen.gov). Besides *gshR*, more than 2-fold reduction was also observed in *gshR*-2 by RealTime-PCR in response to Rex-deficiency (Table 3). Therefore, down-regulation of *gshR's* and *mleSP* could at least in part attribute to the observed defects in tolerance against MV and H_2_O_2_ by TW239 [25].

In *S. mutans*, NADH oxidase (Nox, SMU.1117c) and alkyl hydroperoxidase (AhpC and AhpF by SMU.764 and SMU.765, respectively) have been shown to play a major role in O_2_ metabolism and oxidative stress response [10], [14], [15], [20], [51]. They function to reduce O_2_ to H_2_O (Nox) or H_2_O_2_ (AhpC and AhpF) with the concomitant oxidation of NADH to NAD^+^ [10]. Nox's functionality is dually beneficial to *S. mutans* with alternative routes for NAD^+^ regeneration, contributing to the efficiency of carbohydrates metabolism and for detoxifying deleterious oxygen metabolites [10], [15], [20]. When analyzed by RealTime-PCR, *nox* and *ahpC* were found to be down-regulated by 2.67- and 1.86-fold (*P*<0.01), respectively in TW239 (Table 3). However, no significant differences were measured in expression of *ahpF*. Decreased expression of Nox further supports the notion that Rex optimizes the catabolic pathways, ensuring efficient carbohydrate utilization without needing NADH oxidase to replenish the NAD^+^ pool for glycolysis re-entry. In addition, the down-regulation of these key anti-oxidant house-keepers also provides rationale as to why TW239 has an increased susceptibility to oxidative stress.

Data continues to accumulate, suggesting that development of mature biofilms in *S. mutans* requires coordination of various cellular functions in response to environmental conditions, including acid and oxidative stress responses [1], [39], [52], [53]. The weakened tolerance to oxidative stress can certainly be attributed in part to the observed defects in biofilm formation by TW239, and especially the Rex/GuaA-double mutant, JB314. It is known that acid and oxidative stress induce DNA damages, including formation of abasic sites in DNA [54], [55]. The *guaA* gene is annotated to encode a putative glutamine amidotransferase of Class I family enzyme with a potential role in purine ribonucleotide biosynthesis (www.oralgen.lanl.gov), although genes encode enzymes that catalyze the transfer of the amino group from glutamine in purine ribonucleotide biosynthesis, such as amidophosphoribosyltransferase (SMU.32) and phosphoribosyl-formylglycinamidine synthetase (SMU.30), are clearly identified in an apparent operon. While the exact role of GuaA as well as the rationale for transcriptionally linking *rex* and *guaA* awaits further investigation, elevated expression of enzymes for ribonucleotide biosynthesis in TW239 could partly compensate the need for the repair of DNA damaged by oxidative stress as a result of Rex-deficiency. The additive effects of GuaA- and Rex-deficiency in acid and oxidative stress tolerance and biofilm formation as seen in JB314 further support this notion.

In summary, we further demonstrated that *rex* and *guaA* in *S. mutans* are co-transcribed and auto-regulated. As a redox-sensor, Rex can function as a transcriptional repressor as well as an activator in response to intracellular NADH/NAD^+^ level and metabolic state and plays a major role in regulation of central carbon metabolism, oxidative stress tolerance and biofilm formation. Further investigation is underway to pinpoint the binding site of Rex activator and elucidate the role of GuaA in *S. mutans* cellular physiology.

## Supporting Information

Figure S1 **Rex-DNA interactions are specific.** (A) EMSA stained with SYBR gold showing that Rex binding to the promoter of *adhE* is dependent on highly conserved nucleotides within the Rex-box. When incubated with annealed synthetic DNA oligo 148Fw and 148Rv, which contain the conserved Rex-box of the *adhE* promoter, the recombinant Rex caused mobility shift. As expected, no apparent binding and shift were observed when mixed with oligos with the conserved nucleotides mutated (m148Fw and m148Rv) (Table 2). (B) In a “cold” competition assay, rRex (25 ng) was incubated with a 5′ biotinylated oligo with intact Rex-Box of *adhE* promoter (1 ng) (Table 2) and increasing amounts of the non-biotiny|ated DNA probe. As expected, inclusion of cold probe decreased the mobility shift of the rRex-biotiny|ated promoter complex, as visualized by the LightShift Chemiluminescene EMSA Kit (Pierce, Rockford, IL), and such a effect is dose-dependent. (C) EMSA stained with SYBR gold showing results when Rex was mixed with the promoter of *levD*, which does not contain a Rex-box.
(TIF)

Figure S2 **End-point L-lactate determination.** *S. mutans* strains were grown aerobically in BHI. After 24 hours, L-lactate concentrations in the culture supernates were quantified by using EnzyChrom™ L-Lactate Assay Kit (BioAssay Systems, Hayward, CA). Preliminary results showed that the Rex-deficient mutant, TW239, produced 1.5 mM less lactate than the wildtype, UA159. Similar results were also seen with TW263, a GuaA-deficient mutant, and JB314, a Rex/GuaA double mutant. Complementation of TW239 and TW263 with the respective wild-type copy plus the cognate promoter in shuttle vector pDL278 in strain TW239C and TW263C, respectively, was able to partially restore the wildtype lactate levels.
(TIF)

Figure S3 **Glycolytic pH drop.** *S. mutans* strains were grown to an O.D. of 0.5, washed twice in ice cold water, and resuspended in 50 mM KCI and 1 mM MgCl_2_ before glucose (50 mM) was added to initiate glycolysis as detailed in Materials and Methods. Results showed that relative to the wild-type, UA159 (solid square), the Rex-deficient mutant, TW239 (open triangle), displayed a slower pH drop. Additionally, the resting pH of TW239 after 20 minutes was higher than that of UA159, as can be seen more clearly in the inset. Complementation of the mutant with the wild-type *rex* plus its cognate promoter in shuttle vector pDL278 (TW239C, solid triangle) was able to partly restore the phenotype to the wild-type. Similar trends but in a less degree was also seen with TW263, a GuaA-deficient mutant. TW263C, TW263 carrying shuttle vector pDL278 with the wild-type *guaA*; JB314, a Rex and GuaA-deficient double mutant.
(TIF)

Figure S4 **Hydrogen Peroxide Killing of Anaerobic Biofilms.** *S. mutans* biofilms were grown on glass slides anaerobically in BMGS. After 48 hours, biofilms were harvested, briefly sonicated, and washed with 0.1 M glycine, pH 7.0. The cells were then resuspended with 0.1 M glycine, pH 7.0 containing 0.2% (w/v) and hydrogen peroxide, and incubated for periods of 0, 90, and 110 minutes. At each time point, samples were taken, serially diluted, and plated in triplicate on agar plates. Results showed that mutants deficient of Rex (TW239) and GuaA (TW263) were more than 1-log more susceptible to hydrogen peroxide after 110 minutes, as compared to the wild-type, UA159. Relative to UA159, the Rex/GuaA double mutant, JB314, is 3-logs more susceptible to hydrogen peroxide.
(TIF)

Table S1 **Primers used for RealTime-PCR in this study.**
(PDF)
