# Supplementary figures and images for: The Redox-Sensing Regulator Rex Modulates Central Carbon Metabolism, Stress Tolerance Response and Biofilm Formation by Streptococcus mutans
Source: PLoS One. 2012 Sep 13;7(9):e44766. doi: 10.1371/journal.pone.0044766 (PMC3441419; doi:10.1371/journal.pone.0044766)

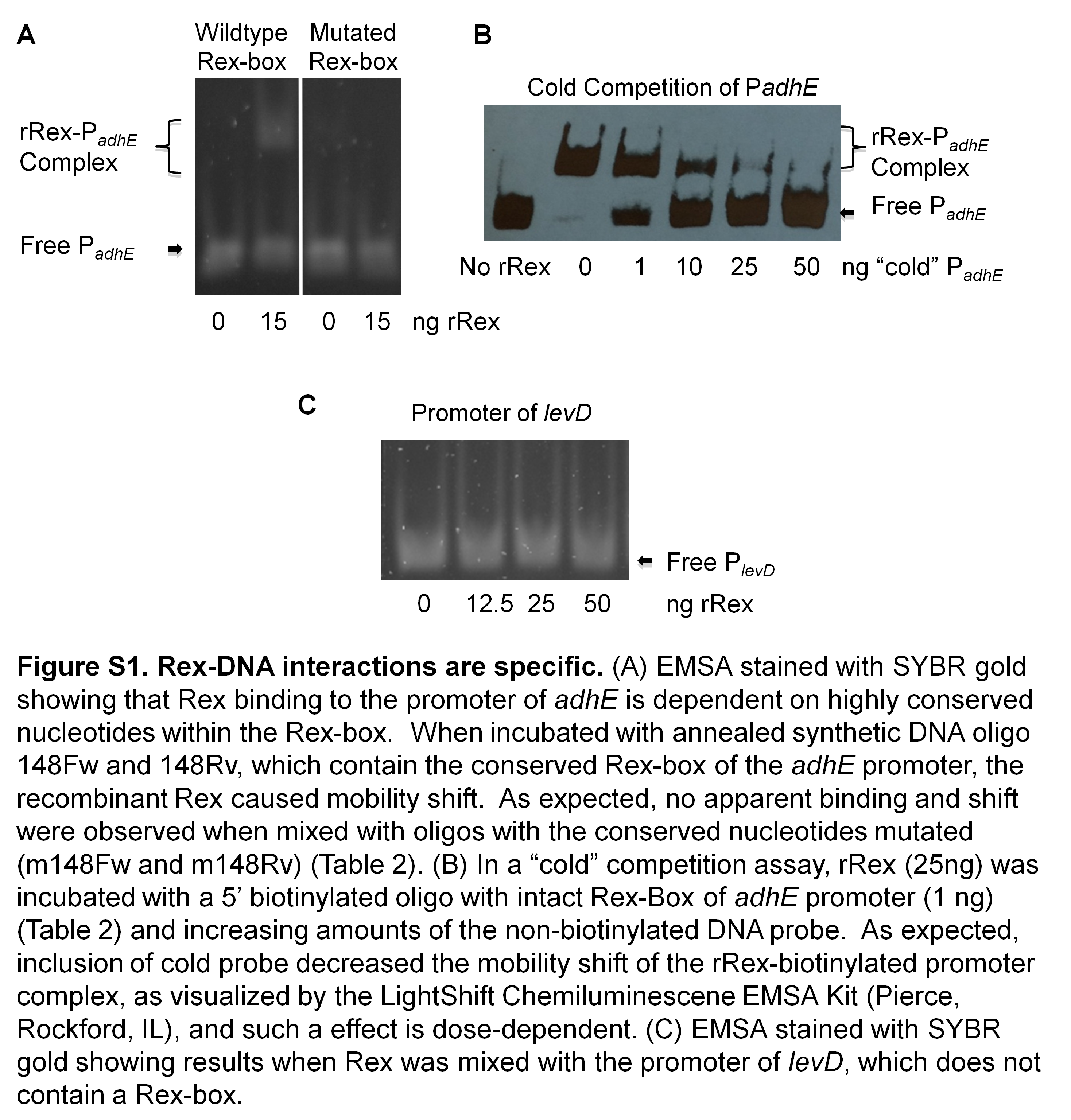

Supplement: Figure S1 — Rex-DNA interactions are specific. (A) EMSA stained with SYBR gold showing that Rex binding to the promoter of adhE is dependent on highly conserved nucleotides within the Rex-box. When incubated with annealed synthetic DNA oligo 148Fw and 148Rv, which contain the conserved Rex-box of the adhE promoter, the recombinant Rex caused mobility shift. As expected, no apparent binding and shift were observed when mixed with oligos with the conserved nucleotides mutated (m148Fw and m148Rv) (Table 2). (B) In a “cold” competition assay, rRex (25 ng) was incubated with a 5′ biotinylated oligo with intact Rex-Box of adhE promoter (1 ng) (Table 2) and increasing amounts of the non-biotiny|ated DNA probe. As expected, inclusion of cold probe decreased the mobility shift of the rRex-biotiny|ated promoter complex, as visualized by the LightShift Chemiluminescene EMSA Kit (Pierce, Rockford, IL), and such a effect is dose-dependent. (C) EMSA stained with SYBR gold showing results when Rex was mixed with the promoter of levD, which does not contain a Rex-box. (TIF) [file pone.0044766.s001.tif]

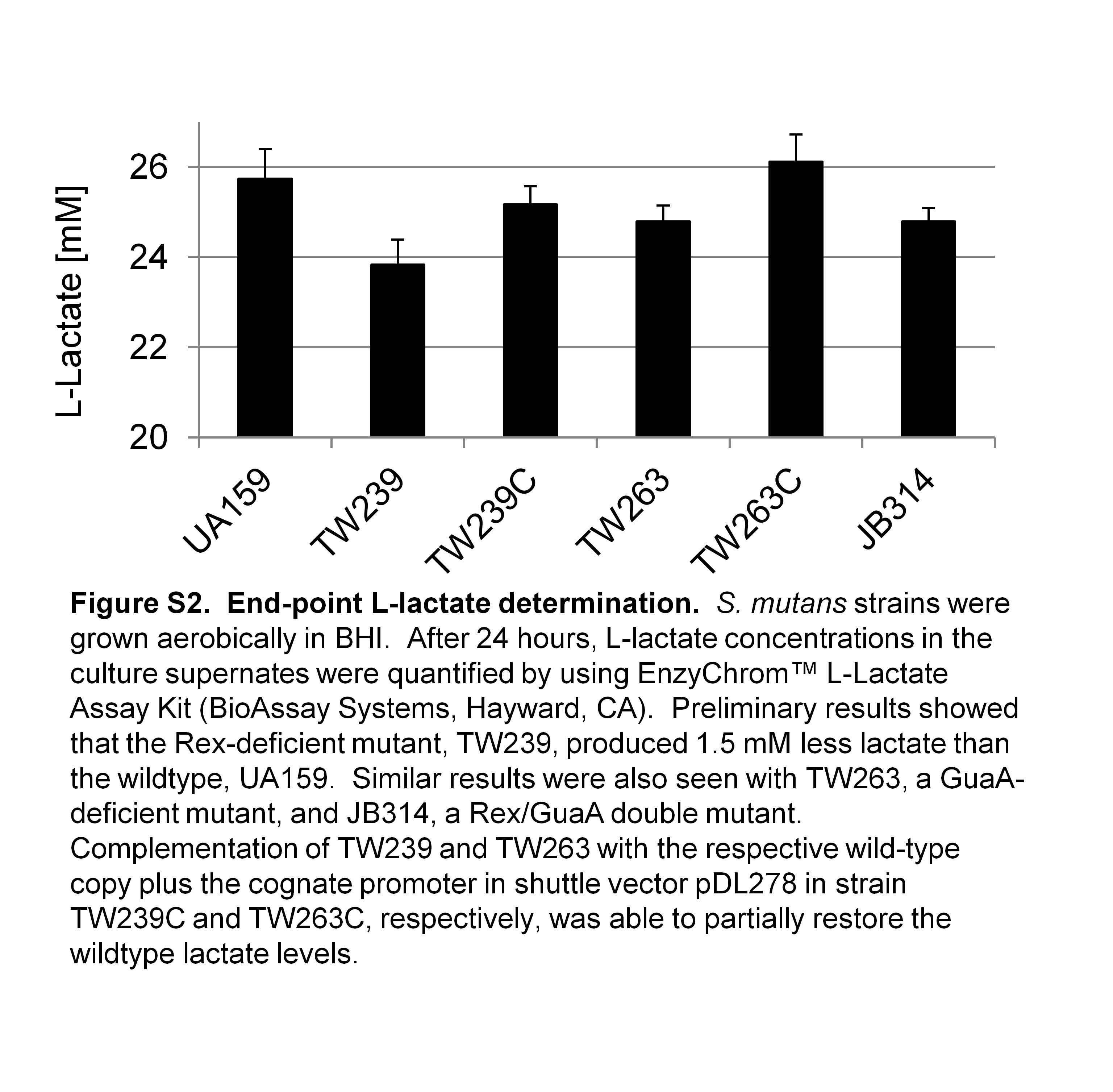

Supplement: Figure S2 — End-point L-lactate determination. S. mutans strains were grown aerobically in BHI. After 24 hours, L-lactate concentrations in the culture supernates were quantified by using EnzyChrom™ L-Lactate Assay Kit (BioAssay Systems, Hayward, CA). Preliminary results showed that the Rex-deficient mutant, TW239, produced 1.5 mM less lactate than the wildtype, UA159. Similar results were also seen with TW263, a GuaA-deficient mutant, and JB314, a Rex/GuaA double mutant. Complementation of TW239 and TW263 with the respective wild-type copy plus the cognate promoter in shuttle vector pDL278 in strain TW239C and TW263C, respectively, was able to partially restore the wildtype lactate levels. (TIF) [file pone.0044766.s002.tif]

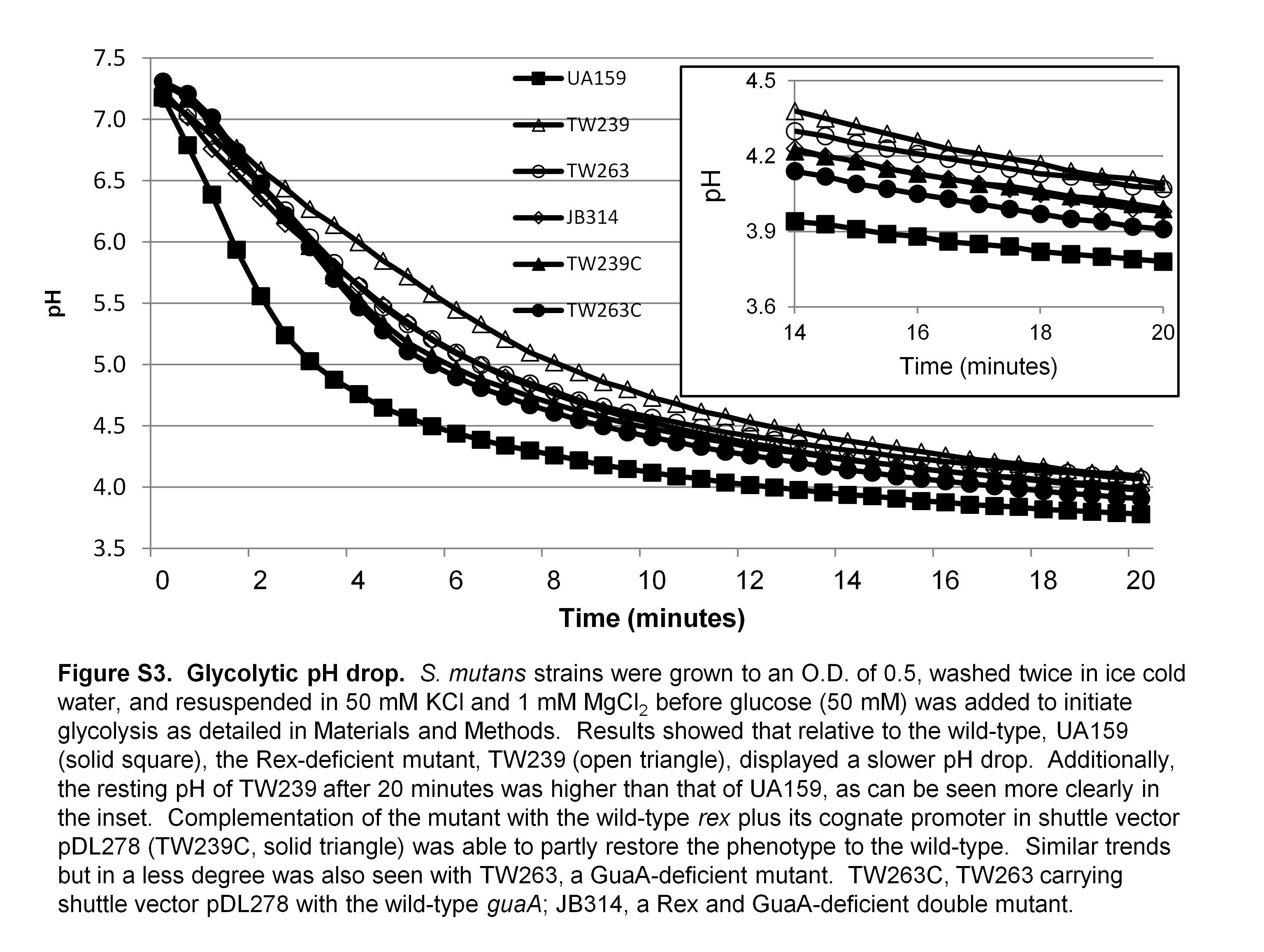

Supplement: Figure S3 — Glycolytic pH drop. S. mutans strains were grown to an O.D. of 0.5, washed twice in ice cold water, and resuspended in 50 mM KCI and 1 mM MgCl2 before glucose (50 mM) was added to initiate glycolysis as detailed in Materials and Methods. Results showed that relative to the wild-type, UA159 (solid square), the Rex-deficient mutant, TW239 (open triangle), displayed a slower pH drop. Additionally, the resting pH of TW239 after 20 minutes was higher than that of UA159, as can be seen more clearly in the inset. Complementation of the mutant with the wild-type rex plus its cognate promoter in shuttle vector pDL278 (TW239C, solid triangle) was able to partly restore the phenotype to the wild-type. Similar trends but in a less degree was also seen with TW263, a GuaA-deficient mutant. TW263C, TW263 carrying shuttle vector pDL278 with the wild-type guaA; JB314, a Rex and GuaA-deficient double mutant. (TIF) [file pone.0044766.s003.tif]

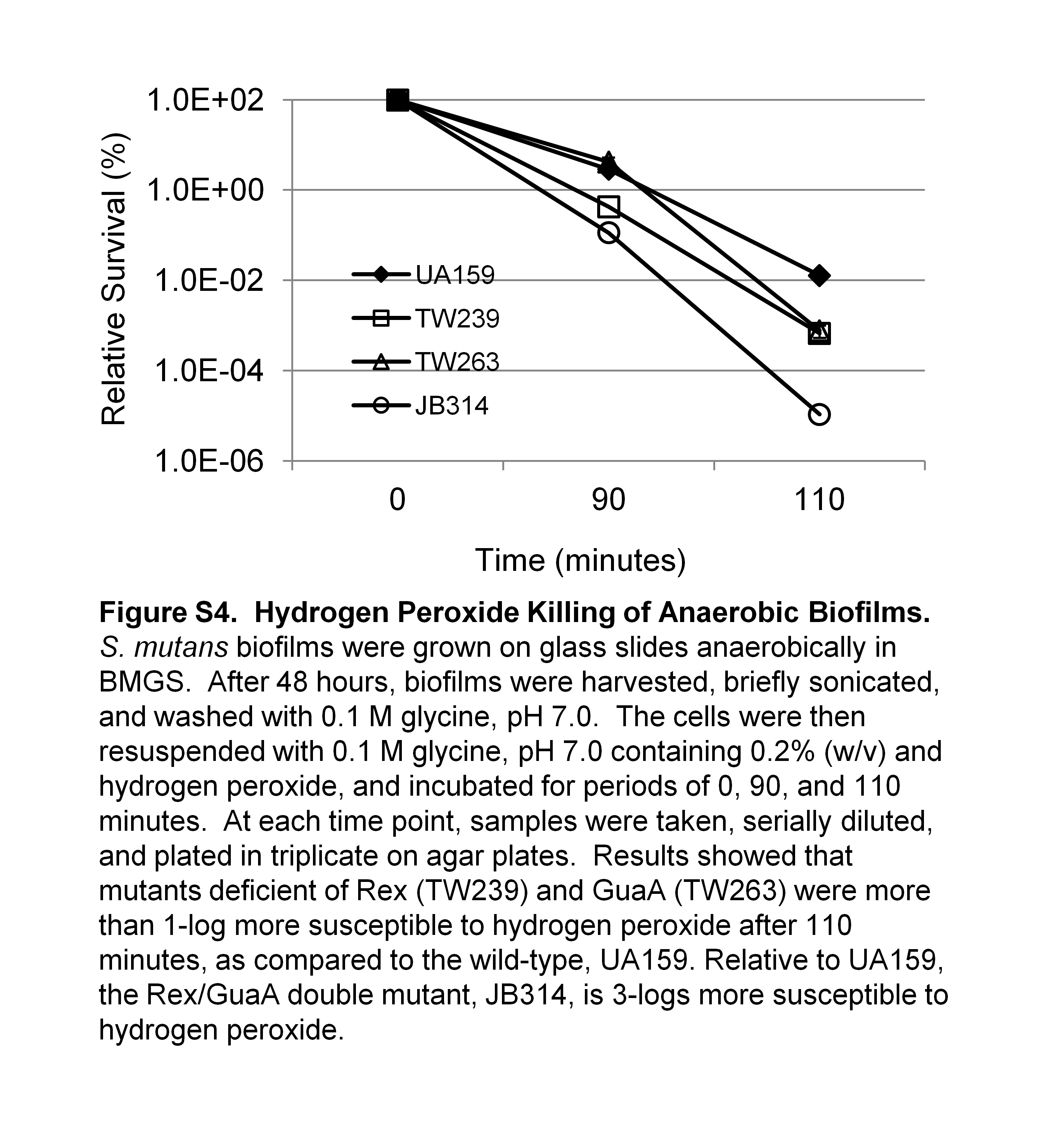

Supplement: Figure S4 — Hydrogen Peroxide Killing of Anaerobic Biofilms. S. mutans biofilms were grown on glass slides anaerobically in BMGS. After 48 hours, biofilms were harvested, briefly sonicated, and washed with 0.1 M glycine, pH 7.0. The cells were then resuspended with 0.1 M glycine, pH 7.0 containing 0.2% (w/v) and hydrogen peroxide, and incubated for periods of 0, 90, and 110 minutes. At each time point, samples were taken, serially diluted, and plated in triplicate on agar plates. Results showed that mutants deficient of Rex (TW239) and GuaA (TW263) were more than 1-log more susceptible to hydrogen peroxide after 110 minutes, as compared to the wild-type, UA159. Relative to UA159, the Rex/GuaA double mutant, JB314, is 3-logs more susceptible to hydrogen peroxide. (TIF) [file pone.0044766.s004.tif]
