# Supplementary material for: The Redox-Sensing Regulator Rex Modulates Central Carbon Metabolism, Stress Tolerance Response and Biofilm Formation by Streptococcus mutans
Source: PLoS One. 2012 Sep 13;7(9):e44766. doi: 10.1371/journal.pone.0044766 (PMC3441419; doi:10.1371/journal.pone.0044766)
Supplement: Table S1 — Primers used for RealTime-PCR in this study. (PDF) [file pone.0044766.s005.pdf]

Table 1S. Primers used for RealTime-PCR in this study

Name	Nucleotide Sequence	Applications
16S RNAFw	Forward, 5’-CACACCGCCCGTCACACC-3’	16S rRNA (160 bp)
16S RNARev	Reverse, 5’-CAGCCGCACCTTCCGATACG-3’	16S rRNA (160 bp)
SMU.140Fw	Forward, 5’- ACTTCAGGCTATCAGGTAA-3’	*gshR* (131 bp)
SMU.140Rev	Reverse, 5’-GCATAGAATATACCGAGCG -3’	*gshR* (131 bp)
SMU.1115Fw	Forward, 5’-TTGGCGACGCTCTTGATCTTAG-3’	*ldh* (92 bp)
SMU.1115Rev	Reverse, 5’-GTCAGCATCCGCACAGTCTTC-3’	*ldh* (92 bp)
SMU.127Fw	Forward, 5’-GGAGACTCGGCAACTAAT-3’	*adhA* (175 bp)
SMU.127Rev	Reverse, 5’-CAGGAATACCATAAGCATCAG-3’	*adhA* (175 bp)
SMU.1117cFw	Forward, 5’-AACACCAATCTTACCACCTA-3’	*nox-2* (171 bp)
SMU.1117cRev	Reverse, 5’-GCACCAACAACAGCAATA-3’	*nox-2* (171 bp)
SMU.1001Fw	Forward, 5’-CAGAATATGAAGCACAGAGTC-3’	*dpr* (90 bp)
SMU.1001Rev	Reverse, 5’-CTACCATAACACCTTGAGATAAC-3’	*dpr* (90 bp)
SMU.764Fw	Forward, 5’-GATGGTATTGGTCGTGATG-3’	*ahpC* (80 bp)
SMU.764Rev	Reverse, 5’-ACTTCTCCTGGATGTTGG-3’	*ahpC* (80 bp)
SMU.838Fw	Forward, 5’-TAACCAGTCACCGTCAAG-3’	*gshR2*, *gor* (97 bp)
SMU.838Rev	Reverse, 5’-TCATCAACACCATAACCAATAC-3’	*gshR2*, *gor* (97 bp)
SMU.1410Fw	Forward, 5’-GATTCACCTCTAACACAACTT-3’	*fdrC* (183 bp)
SMU.1410Rev	Reverse, 5’-TGGCTGCTTCGTATAACT-3’	*fdrC* (183 bp)
SMU.137Fw	Forward, 5’-GTATTGCCTCTCGTGTCT-3’	*mleS* (118 bp)
SMU.137Rev	Reverse, 5’-AAGTCGTCCATATCGTCAA-3’	*mleS* (118 bp)
SMU.138Fw	Forward, 5’-GCTCTGCCTTACTTCCTAA-3’	*mleP* (87 bp)
SMU.138Rev	Reverse, 5’-CTTGCTATCCGTCGTCAT-3’	*mleP* (87 bp)
